# Vienna Letter

**Published:** 1899-03

**Authors:** 


### CORRESPONDENCE.

### VIENNA LETTER.

VIENNA, February 1, 1899.

The radical operation on the mastoid as performed by Dr. Jansen, of Berlin, requires about one-half the time in the healing process as is ordinarily necessary after such operations.

After the mastoid cells, antrum, tympanic cavity and auditory canals are converted into one cavity, and all diseased tissue removed, he prepares the cavity, by smoothing all the rough surfaces with a burr, after which skin-grafting is done after the method of Tiersch.

A flap is made from the posterior canal wall. The skin grafts and flap are held in position by gauze, packed through the auditory meatus, the flap being pressed firmly back in contact with the posterior bony wall, thereby forming also a portion of the outer wall. The original incision is closed with sutures and dressed in the usual manner, getting union by first intention.

The cavity is dressed through the auditory meatus.

This modification appeals to me as being of great value, as it practically does away with the usual unsightly disfigurement, and the entire surface is cuticlized in from four to six weeks.

I saw a number of the operations, and the results were beautiful.

Prof. Urbantschitsch, of this place, believes that all chronic suppurative catarrh of the middle ear, if not improved after six weeks' treatment, should be operated upon.

He goes through the antrum into the tympanic cavity and removes the carious bone, the wound is packed with gauze and healing is by granulation. He says that curetting the tympanic cavity is not sufficient to effect a cure and they finally have to come to the operation through the antrum.

Mr. Arthur Cheattle, of London, and others, claim that most cases of chronic discharges, if not of too long standing, are relieved by the tympanic operation. This I can heartily attest, as I saw a number of patients that had been operated on by this method and the result was all that could be desired.

W. C. WARREN, M.D.

---

MEMPHIS, TENN., February 13, 1899.

DEAR SIR:—At the regular annual meeting of the Tri-State Medical Association of Mississippi, Arkansas and Tennessee, held in Memphis, December 20, 21 and 22, 1898, the following resolutions were adopted:

"WHEREAS, The medical laws of the various States have been so perverted by political influences as to give legislative sanction to grotesque, ignorant and dangerous sects of pretenders and charlatans; and

"WHEREAS, The privileges granted to one of the most outrageous aberrations, namely, the so-called Osteopathy, constitute a disgrace to the States in which the 'osteopathists' are legally entrenched; and

"WHEREAS, A certain William Smith, Osteopathist, having been roundly denounced, together with his sect, by Parke, Davis & Co., and the *Medical Age*, now brings suit against both for $25,000 damages; therefore,

"*Be it declared the sentiment of the Tri-State Medical Association of Mississippi, Arkansas and Tennessee,* That Parke, Davis & Co., and the *Medical Age* are entitled to the sympathy of its members and of all medical practitioners; that we wish and expect them to enjoy a complete triumph in repelling this legal assault; and that wheresoever a powerful house takes a bold stand in opposition to quackery it promotes the interests of legitimate and honorable medicine and the welfare of humanity."

RICHMOND MCKINNEY,
Secretary.

